# Clinical research on postoperative trauma care: has the position of observational studies changed?

**DOI:** 10.1007/s00068-016-0720-3

**Published:** 2016-09-01

**Authors:** D. P. J. Smeeing, R. M. Houwert, M. C. Kruyt, O. A. J. van der Meijden, F. Hietbrink

**Affiliations:** 1 Utrecht Traumacenter, Utrecht, The Netherlands; 2 Department of Surgery, University Medical Centre Utrecht, PO Box 85500, 3508 GA Utrecht, The Netherlands; 3 Department of Surgery, St Antonius Hospital Nieuwegein, Nieuwegein, The Netherlands; 4 Department of Orthopedic Surgery, University Medical Centre, Utrecht, The Netherlands; 5 Department of Orthopedic Surgery, SpaarneGasthuis, Hoofddorp, The Netherlands

**Keywords:** Ankle fracture, Postoperative care, Epidemiology, Research perspective, Study design

## Abstract

**Objective:**

The postoperative care regimes of ankle fractures are studied for over 30 years and recommendations have shifted only slightly in the last decades. However, study methodology might have evolved. The aim of this study was to evaluate the changes in time in the design, quality and outcome measures of studies investigating the postoperative care of ankle fractures.

**Methods:**

The MEDLINE and EMBASE database were searched for both RCTs and cohort studies. The original studies were divided into decades of publication over the last 30 years. The methodological quality of the studies was assessed using the ‘traditional’ risk of bias assessment tool provided by The Cochrane Collaboration and the ‘newer’ MINORS criteria.

**Results:**

The percentage of RCTs on this subject declined from 67 to 38 % in the last decades. According to the Cochrane tool, the reported quality of RCTs has improved in the last three decades whereas the reported quality of observational studies has remained unchanged. However, when quality was evaluated with the MINORS criteria, equal improvement was observed for both RCTs and observational studies. In the 80s, 67 % of all studies used the range of motion as the primary outcome measure, which decreased to 45 % in the 90s. In the 00s, none of the studies used the range of motion as the primary outcome.

**Conclusion:**

For postoperative care of ankle fractures, results of this study showed a relative decrease in the published number of RCTs. The overall quality of the published articles did not decline. In addition, a gradual shift from physician measured to patient-reported outcome variables was observed. However, it should be borne in mind that the findings are based on a small sample (*n* = 25).

## Introduction

In evidence-based medicine, the value of observational studies, such as cohort and case–control studies, has generally been regarded as relatively insignificant [1]. Randomized controlled trials (RCTs) have long been considered the gold standard [2]. In the strive for optimal evidence-based medicine and subsequent patient care, the utility of conducting Randomized Controlled Trials (RCT) in trauma surgery has been questioned over the past years [3]. In addition, observational studies are published frequently, while the number of surgical RCTs from North America has decreased over the last decade [4]. This raises questions how the quality of studies evolved over the last years. In addition, as a result of evolving clinical experience and meeting patient demands, there is more emphasis on patient-reported outcome measurements. Patients are more involved in the decision-making process concerning their treatment, which should ultimately lead to shared decision making.

In trauma surgery, ankle fractures are among the most common fractures and the indication for surgery has not changed in the past decades [5]. Furthermore, the postoperative care regimes of ankle fractures are studied for over 30 years and recommendations have shifted only slightly in the last decades, as we demonstrated recently. As a consequence, the circumstances in which studies were performed did not change substantially in this field of medicine. Therefore, treatment of ankle fractures can be used as an example to investigate the evolution in study methodology. The goal of this study was to evaluate the changes in time in the design, quality and outcome measures of studies investigating the postoperative care of ankle fractures.

## Methods

### Selection

This study included the same studies as selected in our previous meta-analysis evaluating the postoperative care regimen of ankle fractures of twenty-five RCTs and cohort studies [6]. The studies included were retrieved by two reviewers (DS and FH), who independently searched the MEDLINE and EMBASE electronic databases. An extensive description of the search strategy and study selection with inclusion and exclusion criteria was published in the meta-analysis [6].

### Data extraction

The original studies were divided into decades of publication, for the last 30 years. The decades covered the years from 1980 until 1989, 1990 until 1999 and 2000 and onwards. Subsequently, the studies were analysed for design, number of patients, quality, primary outcome variable and other outcomes including complications. The study designs were divided into a retrospective cohort, prospective cohort, randomised controlled trial or a combination of designs. Partly prospective and partly retrospective studies were defined as a mixed cohort study. The methodological quality of the studies was independently assessed by three reviewers (DS, FH and RH) using the risk of bias assessment tool provided by The Cochrane Collaboration and the MINORS criteria [7, 8]. The risk of bias assessment tool provided by The Cochrane Collaboration was developed to compare the quality of RCTs and the MINORS criteria were developed to compare the quality of nonrandomized studies. A higher score indicates a higher quality. In addition, we have checked the affiliations of the authors of the included articles to check if a methodologist cooperated in the research project. The primary outcome variables were the range of motion (ROM), functional scores (such as the Olerud Molander score; a patient-reported outcome) and return to work data [9]. Secondary outcomes ranged from objective physician measured outcomes to more subjective patient-dependent outcomes. If none of the above-mentioned outcome variables were used, the study was excluded from the analysis.

### Descriptive analysis

SPSS software (version 20.0, Chicago, IL, USA) was used for descriptive analysis, both statistically and graphically. Additional analyses were not performed.

## Results

A total of 25 studies were included, 6 of which from the 80s, 11 from the 90s and 8 from the twenty-first century (Table 1). From all studies, 12 (48 %) were RCTs.

**Table 1:** Risk of bias assessment

References	Study design	Positive points on Cochrane risk of bias assessment tool	MINORS criteria	Primary outcomes	Most patient-dependent outcome	All outcomes	Number of included patients
Gul et al. [10]	Retrospective	1	18	RTW	RTW	RTW, FS	50
Honigmann et al. [11]	RCT	4	19	FS	RTW	RTW, FS, ROM	45
Vioreanu et al. [12]	Prospective	2	21	FS	RTW	RTW, FS, ROM	62
Simanski et al. [13]	Mixed	2	18	FS	RTW	RTW, FS	46
Siddique et al. [14]	Prospective	2	17	FS	FS	FS, ROM	44
Lehtonen et al. [15]	RCT	5	21	FS	RTW	RTW, FS, ROM	100
Egol et al. [16]	RCT	3	19	FS	RTW	RTW, FS	55
Harager et al. [17]	Mixed	0	12	ROM	NR	NR	135
Dogra et al. [18]	RCT	5	19	ROM	FS	FS, ROM	52
Laarhoven van et al. [19]	Prospective	2	18	FS	RTW	RTW, FS, ROM	81
Richter et al. [20]	Prospective	1	14	FS	FS	FS	61
Tropp et al. [21]	RCT	2	16	ROM	FS	FS, ROM	30
DiStasio et al. [22]	RCT	3	17	FS	RTW	RTW, FS, ROM	61
Hedström et al. [23]	RCT	4	18	FS	FS	FS, ROM	53
Ahl et al. [24]	Mixed	1	14	FS	FS	FS, ROM	40
Godsiff et al. [25]	Prospective	2	15	ROM	RTW	RTW, ROM	47
Cimino et al. [26]	Mixed	1	12	ROM	ROM	ROM	51
Davies et al. [27]	Prospective	1	18	ROM	RTW	RTW, ROM	41
Wetzler et al. [28]	RCT	2	11	RTW	RTW	RTW, ROM	45
Finsen et al. [29]	RCT	2	19	FS	RTW	RTW, FS, ROM	56
Ahl et al. [30]	Mixed	1	13	FS	FS	FS, ROM	51
Ahl et al. [31]	RCT	2	16	ROM	ROM	ROM	53
Ahl et al. [32]	RCT	3	17	ROM	ROM	ROM	46
Søndenaa et al. [33]	RCT	2	11	ROM	ROM	ROM	43
Lund-Kristensen et al. [34]	Prospective	2	15	ROM	ROM	ROM	28

*RTW* return to work or daily activities, *FS* functional score, *ROM* range of motion, *NR* not reported

### Study design

**Fig. 1:**
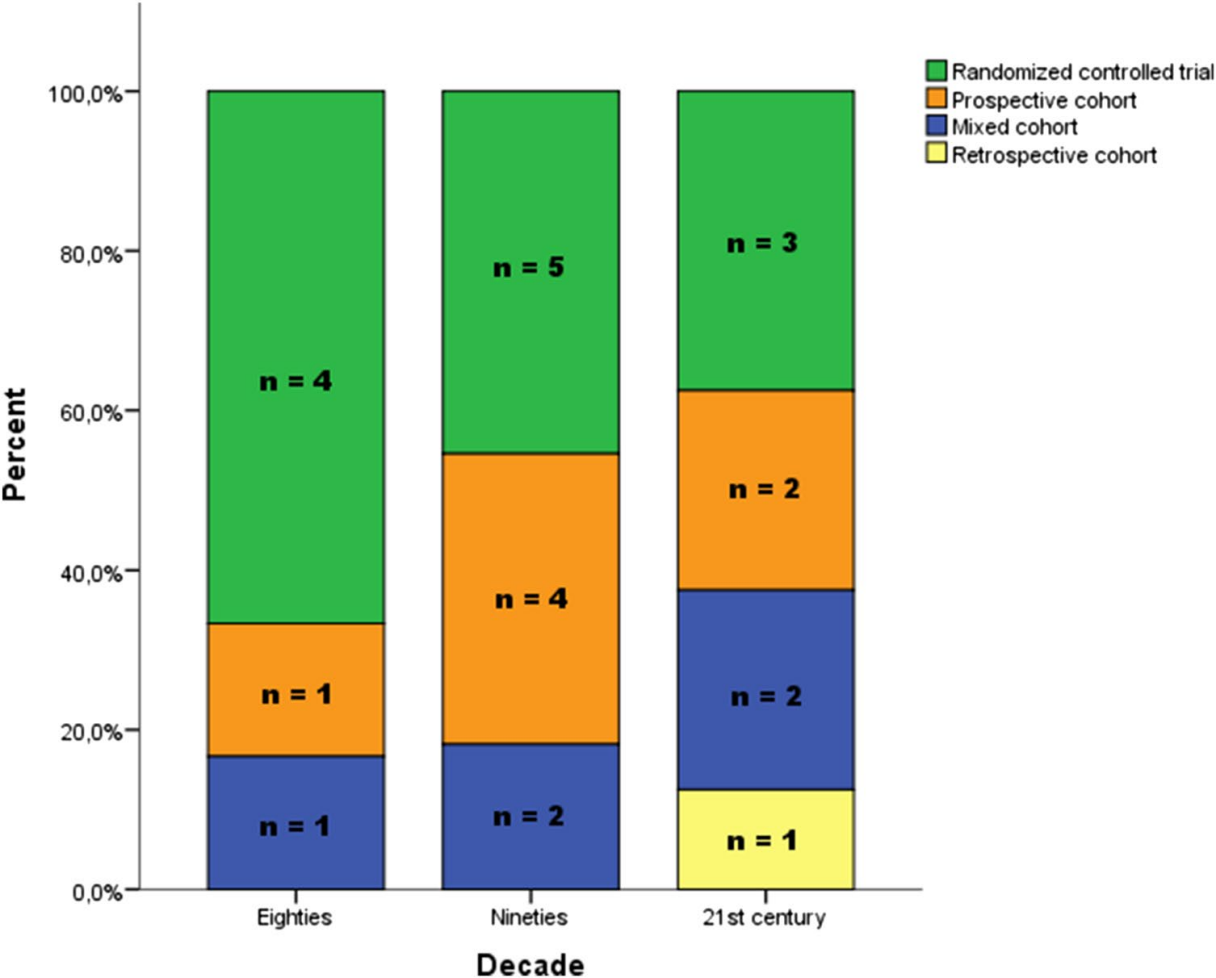
Study design

Fewer RCTs were published in the last decades. The relative number of RCTs decreased. The percentage of RCTs on this subject declined from 67 to 38 % (Table 1; Fig. 1). The number of patients per study changed from 46 (±10) in the 80s to 51 (±13) in the 90s to 67 (±33) in the twenty-first century.

### Quality

**Fig. 2:**
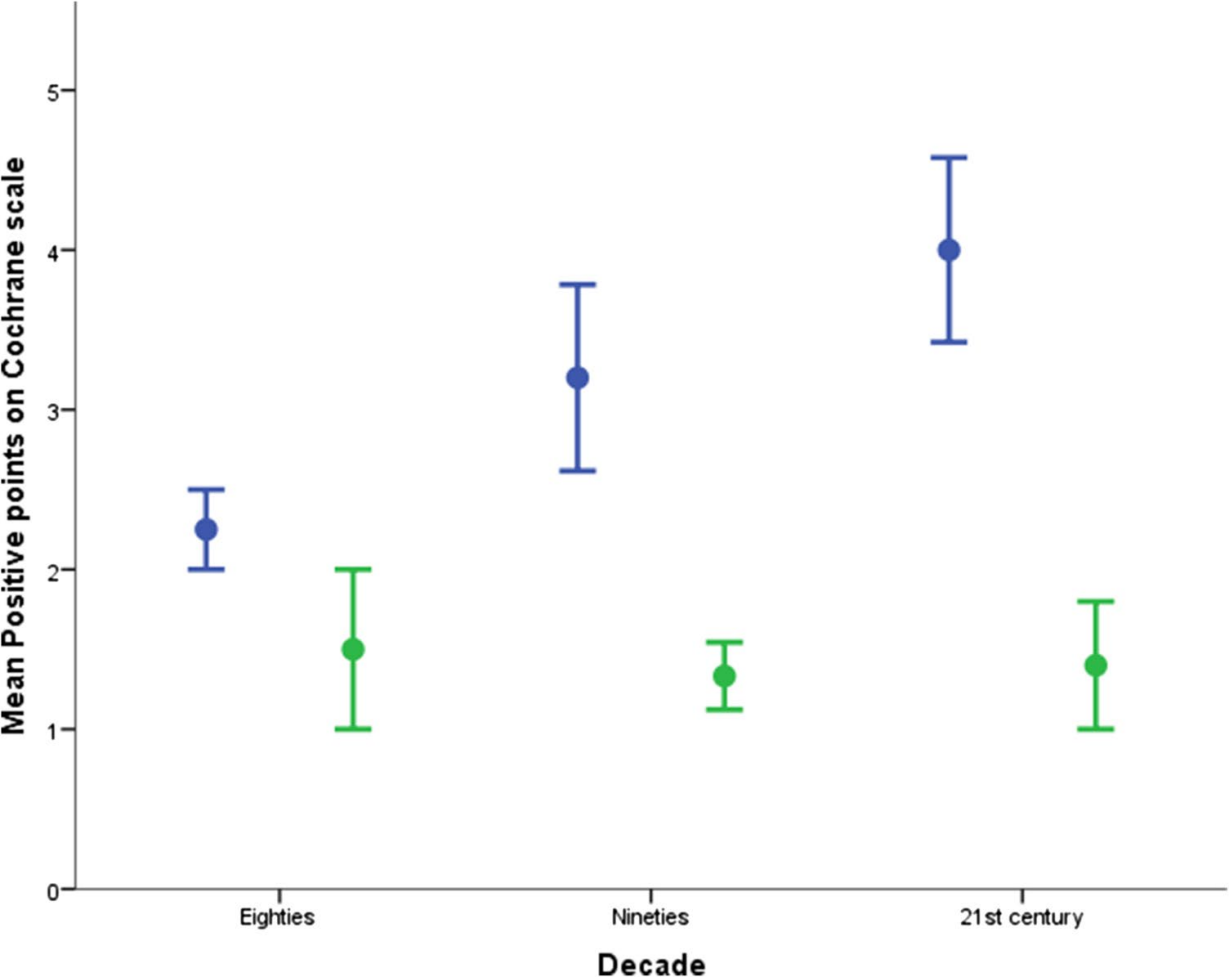
Quality RCT compared with non-randomised studies using Cochrane. *Blue* RCTs, *green* non-randomised studies. *Error bars* ± 1 SE

**Fig. 3:**
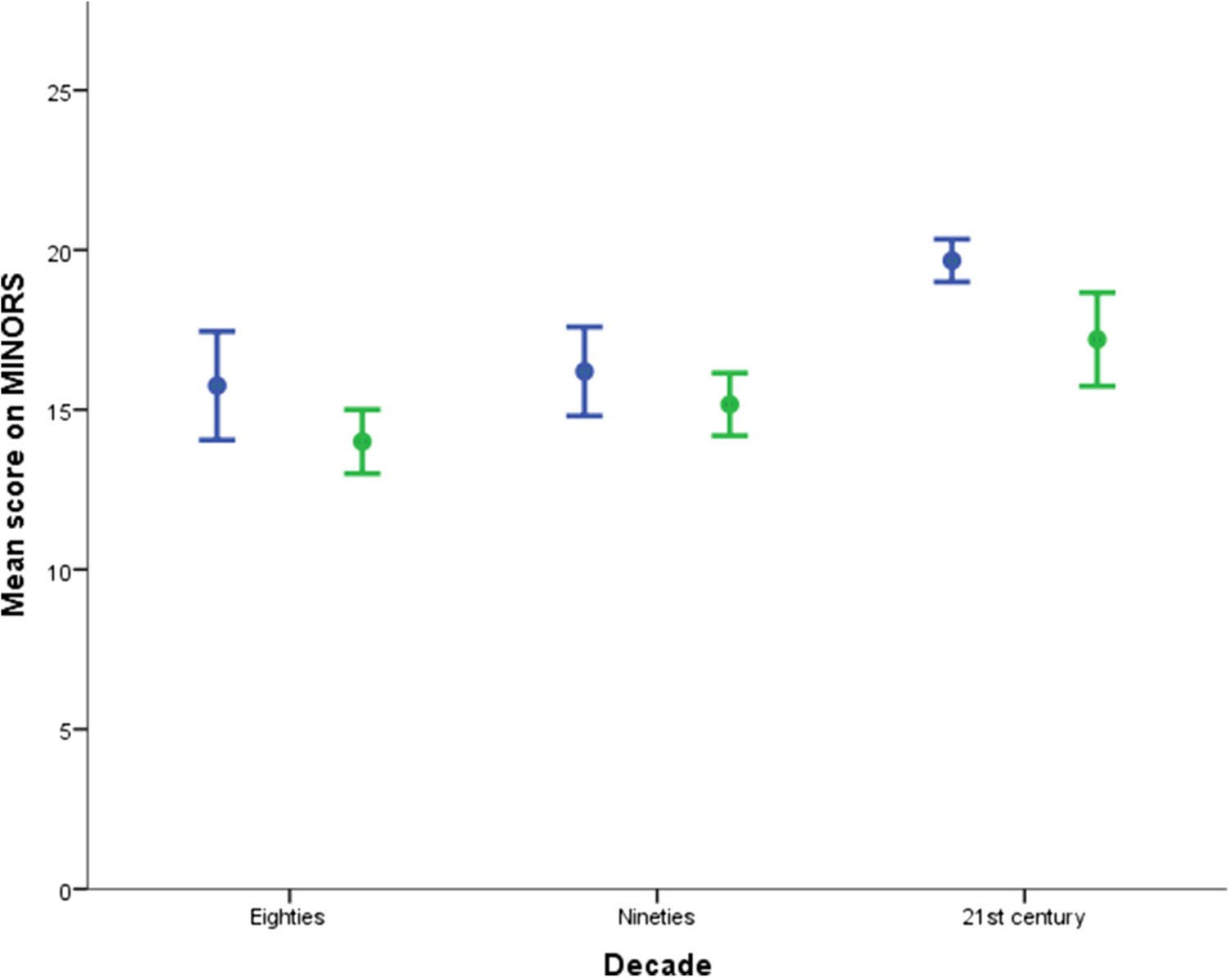
Quality RCT compared with non-randomised studies using MINORS. *Blue* RCTs, *green* non-randomised studies. *Error bars* ± 1 SE

Over the past three decades, the overall quality of the published articles did not decline (Table 1). The mean score on the Cochrane risk of bias assessment tool was 2.2 (±1.3). The highest score was five out of seven points on the Cochrane risk of bias assessment tool, which was reached by two studies. Interestingly, the reported quality of RCTs improved in the last decade, from a mean of 2.3 (±0.5) to 4.0 (±1.0) (Fig. 2). The quality of prospective and mixed cohort studies remained unchanged. The mean score on the MINORS criteria was 16.3 (±2.9). When using the MINORS criteria, the reported quality of RCTs improved in the last decade, from a mean of 15.8 (±3.4) to 19.7 (±1.2). In addition, the quality of prospective and mixed cohort studies improved in the last decade, from a mean of 14.0 (±1.4) to 17.2 (±3.3) (Fig. 3).

Two articles had one or more authors that were affiliated with a research department [11, 18]. Both were RCTs and had a relative high score on the Cochrane risk of bias assessment tool and the MINORS criteria.

### Primary outcome variable and the most patient-dependent outcome variable

**Fig. 4:**
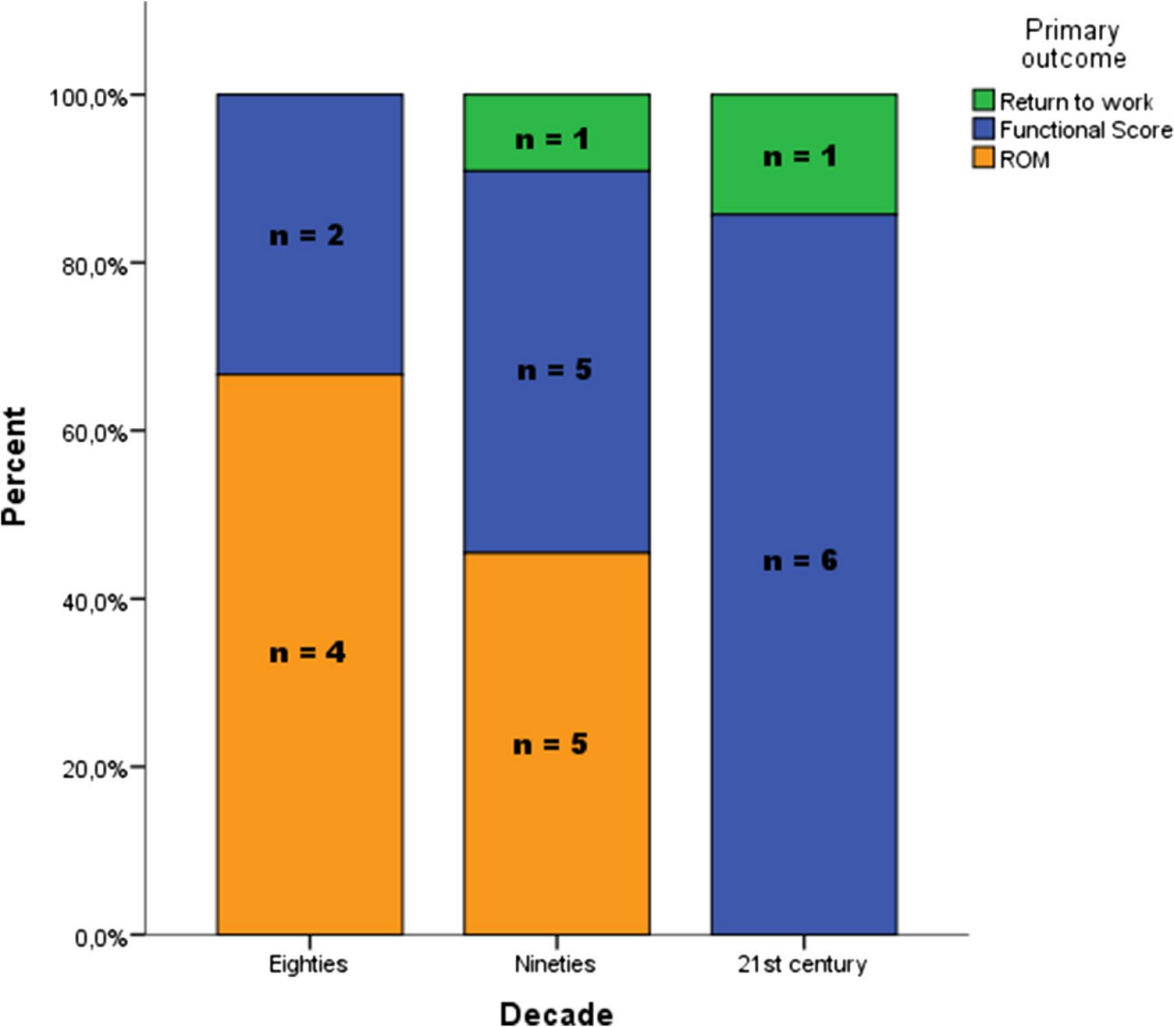
Primary outcomes

A functional score was used as primary outcome in 13 (52 %) studies and the range of motion was used in 10 (40 %) studies as primary outcome. In the 80s, 67 % of all studies used the range of motion as the primary outcome measure, which decreased to 45 % in the 90s. In the 00s, none of the studies used the range of motion as the primary outcome. Less objective outcome measures and more functional scores were used over time (Table 1; Fig. 4).

**Fig. 5:**
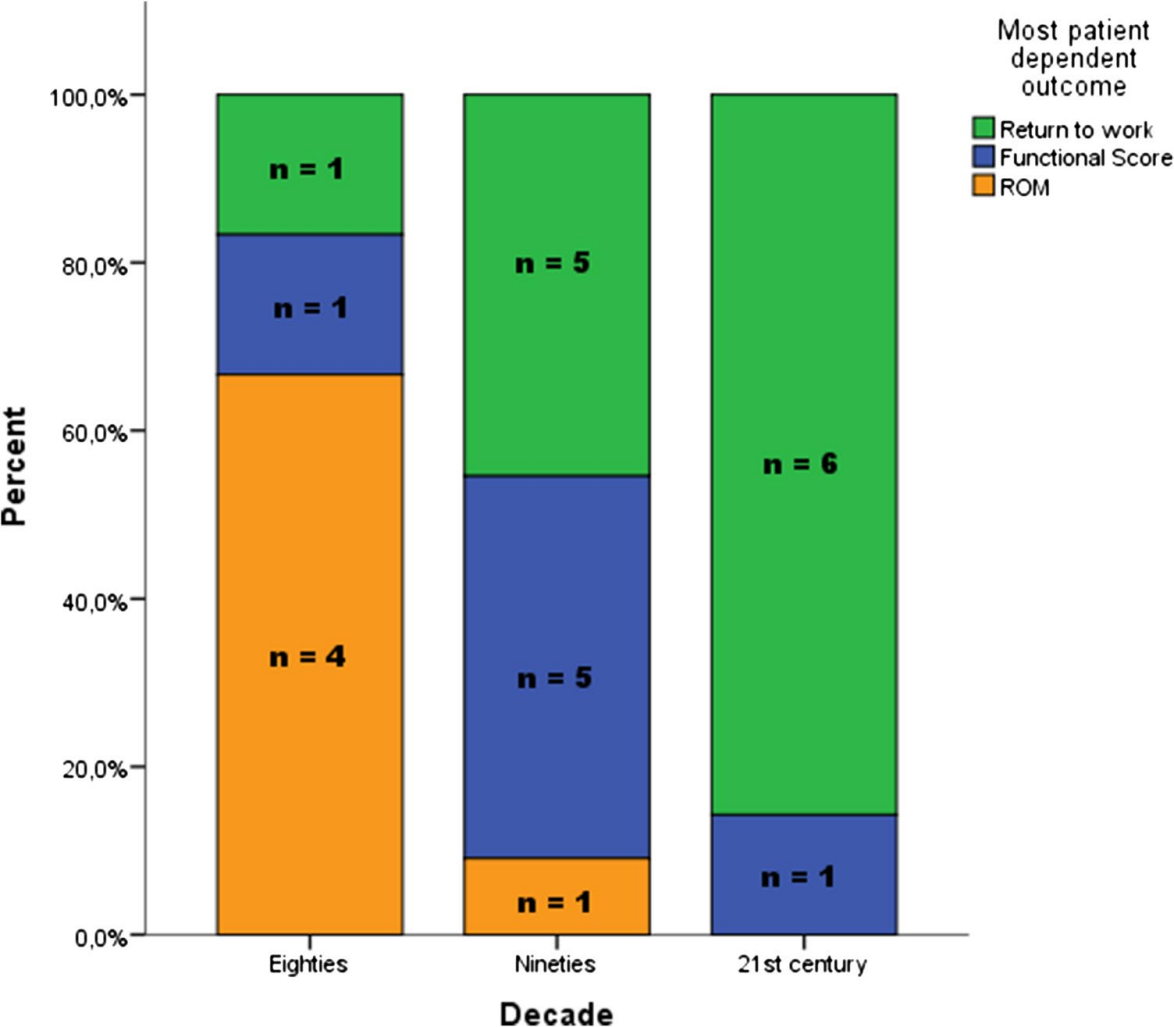
Most patient-dependent outcome

The return to work was the most used patient-dependent outcome variable applied in 12 (48 %) studies (Fig. 5). In the 80s, all six studies used the range of motion as outcome compared to four studies (50 %) in the 00s. In the last decade, six studies (75 %) reported “return to work” data.

A total of two studies did not report on complications [14, 25]. Six other studies did report on complications, but without reporting the treatment group to which the complications belong or without reporting exact numbers [18–24, 26–29, 34].

## Discussion

For postoperative care of ankle fractures, a change in the study design and presented outcome variables was observed over the last three decades. This study demonstrated an increase in the published number of cohort series on the treatment of ankle fractures, with increasing number of patients included. According to the Cochrane tool, the reported quality of RCTs has improved in the last three decades whereas the reported quality of observational studies has remained unchanged. However, when quality was evaluated with the MINORS criteria, equal improvement was observed for both RCTs and observational studies. Therefore, evaluation of quality is under influence of the quality assessment tool used. In addition, a gradual shift from physician measured to patient-reported outcome variables was observed.

There is a growing debate on the need of RCTs to evaluate the effectiveness of surgical procedures [35]. Results of this study indicate a relative decrease in the number of published RCTs over the past three decades in this field of research. Literature shows that one in five surgical RCTs is discontinued early, and one in three completed trials remains unpublished [35]. For (trauma) surgical research, such a trend might be explained by several factors.

First, including patients in a surgical RCT has become more and more challenging [36]. Both patient and surgeon have treatment preferences and surgeons may be better skilled in one of the study procedures. Ideally, randomisation distributes any unknown factors, thereby eliminating unforeseen confounders. However, patients may refuse or prefer the “new” technique and, therefore, do not wish to receive random treatment [37]. In addition, blinding of both patient and surgeon is frequently impossible and/or undesired by both patient and surgeon, which also neutralizes one of the advantages of an RCT over a cohort series.

Second, in the last decade the bureaucratic burden of ethical committee approval and monitoring has increased substantially, especially for conducting RCTs. This is accompanied by an increased financial demand. These procedures were developed to increase patient consent and safety, but sometimes might seem undue. For research projects in a field with limited financial resources, it is therefore increasingly difficult to meet the intensified quality demands [36]. Only a limited amount of funding went to surgical research in spite of the relatively large contribution of surgery to effective treatment [38]. These practical obstacles might have caused a substantial decrease in the quantity of RCTs in surgical research compared to other fields of medicine [4, 39].

However, as the goal of research remains to improve the quality of care for patients with an ankle fracture, alternatives are sought. This might reflect the subsequent increase in the number of published cohort series. An alternative explanation might be that due to the eagerness to produce papers in the highly competitive scientific world, where the number of publications counts heavily, there may be a bias towards writing more relatively inexpensive articles that describe cohort and retrospective studies. However, this was not demonstrated by the absolute number of published papers over the last three decades in this study, nor by a decrease in quality.

Reported quality of RCTs improved over time. This might be explained by several quality assessment tools that became available for RCTs in the 90s. The introduction of these tools provided guidance for constructing well-designed studies, with more standardised reporting and thereby improved reported research quality. Reporting of studies might have improved by reporting standard such as the CONSORT [2]. In the current study, we used the Cochrane risk of bias assessment tool to assess the quality of the studies included. This tool was primarily designed for RCTs, but is also recommended for quality assessment of observational studies [40, 41]. In addition, we used the MINORS criteria [8]. This tool is primarily designed for non-randomized studies. A combination of both tools can provide the overall quality of studies. Due to the design of the Cochrane risk of bias assessment tool, the design of a study is decisive for the results on a domain of the tool (e.g., a randomised trial has the potential to score positive on all domains contrary to observational studies). Therefore, observational studies will inevitably have a higher risk on bias in the Cochrane risk of bias assessment tool. In addition, we used the MINORS criteria [8]. This tool is especially designed for non-randomized studies. Other tools for observational studies were designed more recently, which are not frequently used [42]. It should always be kept in mind that evaluation of quality is under influence of the quality assessment tool used. Similarly to RCTs, the MINORS criteria might provide guidance for constructing well-designed observational studies and further improve observational research quality. Perhaps both RCTs and cohort series are needed to provide a complete image of a disease or treatment modality. RCTs are designed to compare specific items head to head, while large cohort series are more capable of exposing rare complications or the effect of a whole package [43].

A convincing finding of the present study is the increased use of patient-relevant outcomes. The next step would perhaps consist of consensus recommendations to define standard outcome measures for future ankle fracture studies. If all studies applied the same set of outcome measures at the same timepoints, this would make it much easier to compare and to use study results. In rheumatology, such an initiative (called OMERACT) was highly successful [44].

In the 80s, primary outcomes were focused on radiological results and objective variables. The attending physician or investigator could measure these objective variables. In the twentieth century, outcomes increasingly focused on the implications for the patient and were reported as primary end-point of several studies. In the last decade, over half of the included studies mentioned return to work as outcome measure which is, in our opinion, a legitimate patient-reported end-point.

The sample (*n* = 25 studies) is quite small. It is still possible that some of the observed results are attributable to the play of chance. This should be borne in mind when interpreting the results.

As ankle fractures are represented in the top five of most prevalent fractures and have an important socio-economic impact on both patient and society, the observations made in this study might be applicable to broader areas of (trauma) surgery research [45]. However, results of this study might not be applicable to other non-surgical specialties. This study can be seen as a case report and should be tested in further extent and on a larger scale in trauma surgical studies.

Results of this study indicate a relative decrease in the number of published RCTs, which might be a reflection of the practical difficulty to conduct RCTs in trauma surgery due to surgeon, patient and system-based factors. Furthermore, the desire for more patients orientated outcome variables is reflected in the changes seen in the last decades.

## Conclusion

For postoperative care of ankle fractures, a change in study design and presented outcome variables was observed over the last three decades. RCTs were performed less frequently. According to the Cochrane tool, the reported quality of RCTs has improved whereas the reported quality of observational studies has remained unchanged. When quality was evaluated with the MINORS criteria, equal improvement was observed for both RCTs and observational studies. This shows that evaluation of quality is influenced by the quality assessment tool. In addition, a gradual shift from physician measured to patient-reported outcome variables was observed. However, it should be borne in mind that these findings are based on a small sample (*n* = 25).
